# Google Search Trends to assess public interest in and concern about Vuity for treating presbyopia

**DOI:** 10.1371/journal.pone.0293066

**Published:** 2023-10-26

**Authors:** Taku Wakabayashi, Hana A. Mansour, Robert M. Abishek, Jayanth Sridhar, Michael N. Cohen, David Xu, Jordan Deaner, Yoshihiro Yonekawa, Jason Hsu, Ajay E. Kuriyan

**Affiliations:** 1 Wills Eye Hospital, Mid Atlantic Retina, Thomas Jefferson University, Philadelphia, Pennsylvania, United States of America; 2 Bascom Palmer Eye Institute, University of Miami Miller School of Medicine, Miami, Florida, United States of America; Alexandria University Faculty of Medicine, EGYPT

## Abstract

**Purpose:**

To assess public awareness, interest, and concerns regarding Vuity (1.25% pilocarpine hydrochloride ophthalmic solution), an eye drop for the treatment of presbyopia, based on Google Trends.

**Methods:**

We used Google Trends that provides a relative search volume for queried terms, to evaluate searches for Vuity from June 30, 2021, to June 30, 2022, in the United States. The data for this study were downloaded on June 30, 2022. Main outcome measures were changes in relative search volumes for the terms “Vuity,” “Eye drops for reading,” “Eye drops for near vision,” “Presbyopia,” “Pilocarpine,” and related popular search terms, such as “Vuity side effects,” and “Vuity retinal detachment”.

**Results:**

Since the approval of Vuity on October 29, 2021, notable increases in the relative search volumes occurred for Vuity in October 2021, December 2021, and from March to April 2022, which coincided with its approval, availability, and subsequent direct-to-consumer advertising based on positive results of clinical trials. The direct-to-consumer advertising had the greatest impact on the search volume for Vuity. Specific interests included Vuity cost, where to buy it, and its side effects. Retinal detachment was the most highly searched Vuity side effect. Geographic variation was evident, with the relative search volumes highest for “Vuity” in Wyoming, followed by North Dakota.

**Conclusion:**

Google Trends is a useful tool for monitoring increases in public interest in Vuity for presbyopia. Public concerns regarding side effects warrant further real-world investigations of the causal relationship between Vuity and retinal detachment.

## Introduction

Presbyopia is an age-related impairment in near vision that affects 25% (1.8 billion) of the world’s population and at least 114 million people in North America [1]. Vuity (1.25% pilocarpine hydrochloride ophthalmic solution, Allergan, Dublin, Ireland) is the first topical treatment for presbyopia and was approved by the United States Food and Drug Administration (FDA) in October 29, 2021 [2, 3]. Vuity’s theoretical mechanism of action includes contraction of the pupillary sphincter and miosis, resulting in improvement of near vision [2]. The phase 3 randomized clinical trials GEMINI 1 and GEMINI 2 confirmed the efficacy of Vuity in improving near visual acuity in a total of 750 patients aged 40 to 55 years [2]. The most common adverse events were headache and conjunctival hyperemia, with no other serious adverse events. However, topical pilocarpine has long been used at various concentrations for glaucoma, especially since its approval by FDA in 1974, and numerous studies have shown the potential association between pilocarpine and rhegmatogenous retinal detachment (RD), although the exact cause–effect relationship remains unclear [4–6]. As expected, the recent real-world experience of Vuity has identified four patients (five eyes) who developed rhegmatogenous RD after Vuity use according to PubMed search (as of October 5, 2022) [7, 8]. Since Vuity is now available by prescription in pharmacies across the US for the general population and not only patients with glaucoma, more people will be using topical pilocarpine. Consequently, it is important for all optometrists and ophthalmologists who prescribe Vuity to understand the public interest and concerns regarding Vuity and related topics in order to give appropriate information to patients when prescribing it.

Online search engines, such as Google, are major sources of information [9]. Analysis of users’ online search behavior can provide a good understanding of public awareness, interest, concerns, and information needs regarding specific health conditions and medications [10]. Google Trends is a publicly available tool for analyzing such online search data and has been used to study a wide variety of topics, including COVID-19 pandemics [11, 12], influenza epidemics [13], conjunctivitis epidemics [14], and cataract surgery [15]. Thus, Google Trends is expected to provide insight into public interest regarding Vuity at the general population level. This insight may then improve the understanding of public information demands regarding Vuity.

The purpose of this study was to use Google Trends to evaluate public awareness, interest, concerns, and information needs regarding Vuity since its approval.

## Materials and methods

### Data acquisition

The institutional review board of the Wills Eye Hospital determined this study to be Non-Human Subjects Research (NHSR) and was therefore exempt from IRB review. Google Trends provides data related to the Google search engine, which accounts for approximately 88% of the total search volume in the United States as of July 2021 [9]. Google Trends provides a relative search volume (RSV), with results shown on a scale ranging from 0 to 100, where 100 corresponds to the peak in search volume within the specified time frame.

To assess the Google Trends data related to Vuity, we used “United States” as the search range, “6/30/2021-6/30/2022” as the time range, “All categories” as the category, and “Web Search” as the search type. We also used “Google shopping” as a search type to evaluate people’s interest in purchasing Vuity. The data for this study were downloaded on June 30, 2022. We queried Google Trends for the following keywords: “Vuity,” “eye drops for reading,” “eye drops for near vision,” “Pilocarpine,” “Presbyopia,” and popular Vuity-related terms, such as “Vuity costs” and “Vuity side effects,” appearing in Google Trends.

Since Google Trends provides an RSV but not the actual number of total searches, we also used the Google Ads Keyword Planner to evaluate the range of total search numbers.

### Geographic variation in the United States

We assessed the geographical variation of the search query “Vuity” and “Presbyopia” across the United States by comparing the RSV for each query between geographic regions (states). The region with the highest search volume was assigned an RSV of 100, and other geographic regions had RSV scores of 0 to 100, depending on their search volumes relative to the highest state. The RSV for each query term in each region was evaluated using the Choropleth maps of Google Trends.

### Analysis of the posts related to Vuity in Reddit and Facebook

To analyze the public concerns about side effects related to Vuity, we analyze the threads in social media, such as Reddit and Facebook. We evaluated the number of threads regarding the serious side effects related to Vuity.

### Statistical analysis

Linear regression analysis was performed using SigmaStat^®^ software (version 4.0) (SPSS Inc., Chicago, USA) to evaluate the correlation between the weekly RSVs (52 different RSVs ranging from 0 to 100 over a 12-month period) of Vuity and related terms. To evaluate the geographical variation in the search volume in the United States, we created the heatmap using Adobe Illustrator 2022. Statistical significance was set at a p-value of < 0.05.

## Results

### Overall web search volume for Vuity

**Fig 1:**
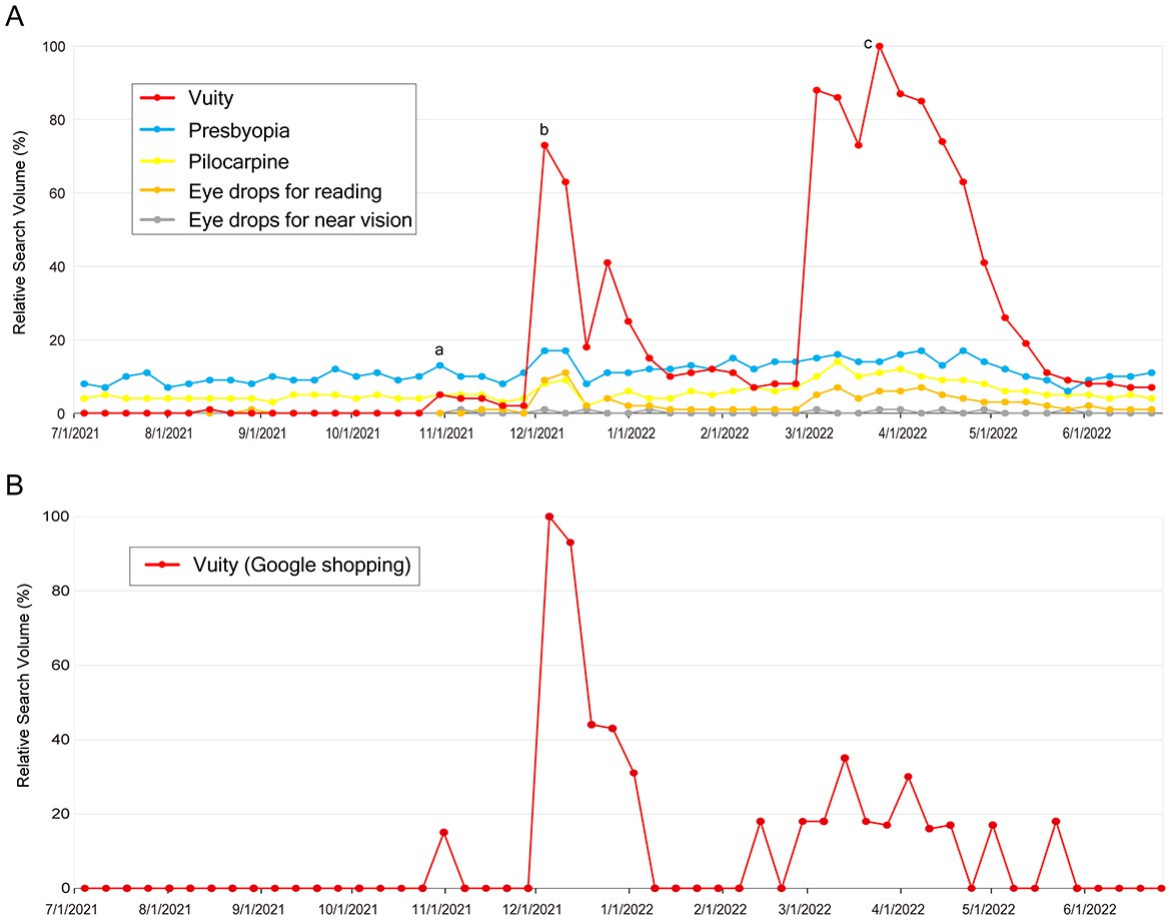
A. Weekly Variation of Overall Search Volumes for “Vuity,” “Presbyopia,” “Pilocarpine,” “Eye drops for reading,” and “Eye drops for near vision” from July 2021 to June 2022 in the United States. a. October 2021: The US Food and Drug Administration approved Vuity for the treatment of presbyopia. b. December 2021: Allergan announced the availability of Vuity. c. March to April 2022: Direct-to-consumer advertising in the form of television commercials was initiated by Allergan, based on the positive results of clinical trials (GEMINI1) published online in March. Allergan also officially announced the results of clinical trials in April 2022. B. Weekly Variation of Search Volumes for “Vuity” in Google shopping.

Prior to the approval of Vuity by the FDA on October 29, 2021, searches for Vuity were rarely conducted, with an RSV of 0 or <1 (Fig 1A). After its approval, a first rise in the RSV level occurred, with an RSV of 4. A second rise then corresponded to the time when Allergan announced the availability of Vuity on December 9, 2021, and represented a 16.8-fold increase (RSV of 67) from the first rise in searches for Vuity. A subsequent third rise with an RSV of 100 then occurred in March 27–April 2, 2022, and represented a 25.0-fold increase from the first rise. This third rise seemed to be influenced by direct-to-consumer advertising (DTCA) in the form of television commercials that were initiated on March 8, 2022 by Allergan, based on the positive results of clinical trials (GEMINI 1) published online on March 3, 2022. Allergan also officially announced the results of clinical trials on April 5, 2022, and there were sustained searches for Vuity in April 2022.

Searches for “eye drops for reading” and “Presbyopia” peaked at the time of availability, while “eye drops for near vision” and “Pilocarpine” peaked at the time of DTCA. A positive correlation was observed in the RSV between “Vuity” and “eye drops for reading” (R^2^ = 0.783, P<0.001), between “Vuity” and “eye drops for near vision” (R^2^ = 0.429, P<0.001), between “Vuity” and “Presbyopia” (R^2^ = 0.325, P<0.001), and between “Vuity” and “Pilocarpine” (R^2^ = 0.779, P<0.001).

The Google Ads Keyword Planner showed a monthly number of searches for “Vuity” of 10,000 to 100,000; therefore, the total number of searches ranged from 120,000 to 1,200,000 during one year of our study period.

### Google shopping search volume for Vuity

Although Vuity cannot be purchased by Google shopping, we evaluated people’s interest in purchasing Vuity by evaluating the search volume of Google Shopping for Vuity. Similar to the overall web search, the search volume of Google Shopping for Vuity showed increases that corresponded with Vuity’s approval, availability, and DTCA (Fig 1B). However, the peak RSV was detected at the time of availability before DTCA. A significant correlation was noted between the overall search volume and the search volume of Google Shopping (R^2^ = 0.254, P<0.001).

### Popular search terms related to Vuity

The popular search terms related to Vuity during the study period included “vuity drops” (popularity: 100), “vuity eye” (99), “vuity eye drops” (97), “vuity cost” (16), “vuity eye drops cost” (10), “vuity reviews” (5), “buy vuity eye drops” (2), “vuity price” (2), “where to buy vuity” (2), “does vuity work” (2) “vuity side effects” (2), “vuity eye drops where to buy” (2), “vuity eye drops price” (2), “how does vuity work” (1), “eye drops for reading vuity” (1), “what is vuity” (1), “vision eye drops vuity” (1), and “vuity stock” (1).

### Search volume for Vuity and side effects

**Fig 2:**
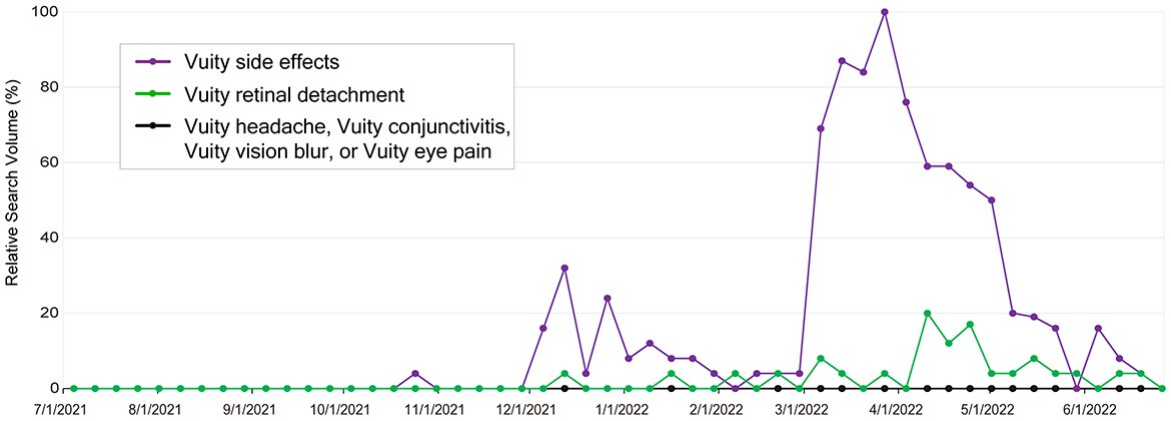
Weekly variation of overall search volumes for “Vuity side effects,” “Vuity retinal detachment,” and “Vuity headache,” “Vuity conjunctivitis,” “Vuity vision blur,” or “Vuity eye pain,” from July 2021 to June 2022 in the United States.

Because the term “vuity side effects” was included in the popular search related to “Vuity,” we evaluated the RSV of the search term “vuity side effects.” Similar to the search term “Vuity,” notable increases in the RSV occurred for “vuity side effects” after FDA approval, availability, and DTCA (Fig 2). We then evaluated the search volume regarding the potential side effects of Vuity. Several side effects, including headache, conjunctival hyperemia, vision blur, and eye pain, reported in the GEMINI 1 and 2 studies did not show any popularity in combination with Vuity. Indeed, the search for “vuity headache,” “vuity conjunctival hyperemia,” “vuity pink eye,”“vuity conjunctivitis,”“vuity blurred vision,” “vuity vision blur,” “vuity hives,” and “vuity eye pain” showed no RSV. However, the search for “vuity retinal detachment” was popular, with an RSV of 4 at the time of Vuity availability, and this term peaked with an RSV of 20 in April 10–16, 2022. Although the RSV of “vuity retinal detachment” was lower than that of “vuity side effects,” retinal detachment was the primary concern among the various potential side effects.

### Geographical variation in the United States

**Fig 3:**
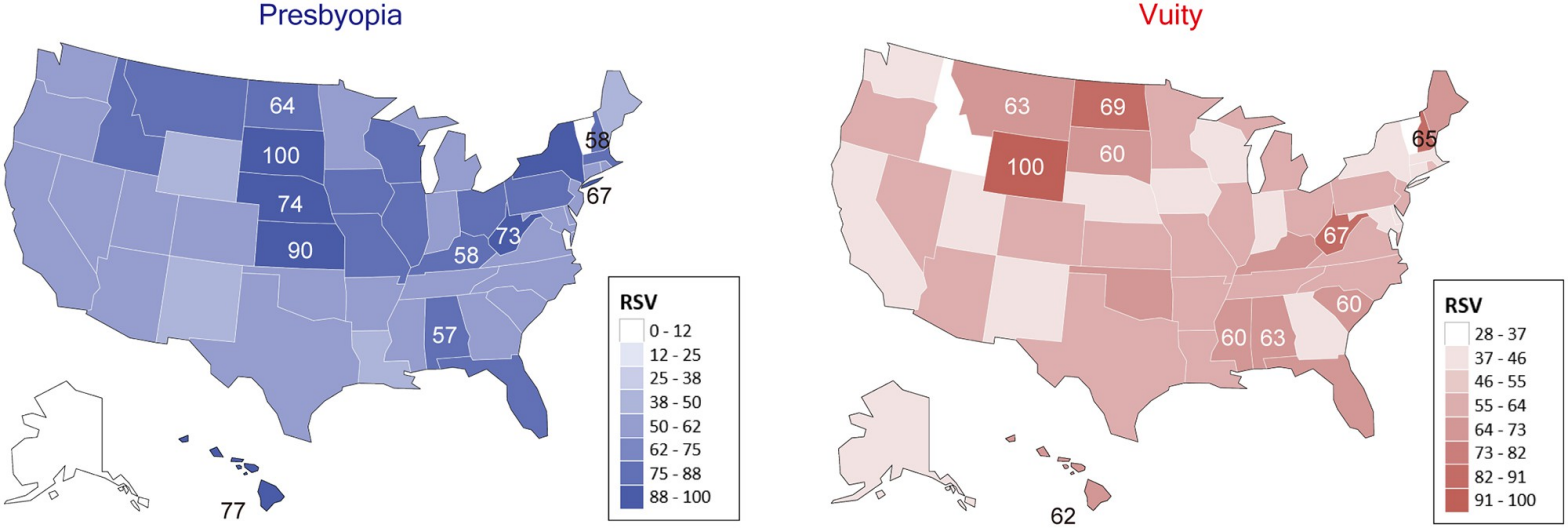
Choropleth maps of Google Trends showing geographic regional differences in interest in presbyopia and Vuity. The top 10 states with the most relative search volume for each search term are listed.

The search volume in each state in the United States showed that residents of Wyoming, North Dakota, and West Virginia searched most frequently, whereas residents of Vermont, the District of Colombia, and Idaho searched least frequently. Wyoming residents searched approximately four times as frequently as residents of Vermont, the state with the fewest relative searches for Vuity. A strong correlation was observed between Vuity and presbyopia searched in each state (R^2^ = 0.911, P<0.001), indicating that states with high searches for presbyopia also had a high number of Vuity searches (Fig 3).

### Analysis of the posts related to Vuity in Reddit and Facebook

Since the RD was likely the primary concern among the various potential side effects related to Vuity, we analyzed the threads on Reddit and Facebook regarding RD associated with Vuity. In Facebook, there was a public group for Vuity users to discuss their experiences. This public group included 272 posts, 1,161 comments, and 1042 likes to the posts. Five (2%) posts talked about RD, and two patients who suffered from RD after Vuity posted their experiences. In Reddit, 33 threads included the word "Vuity," 2 of those threads talked about RD, and one of them was a shared thread about a patient who developed RD described in Facebook.

## Discussion

This study evaluated the online search behavior in the United States related to Vuity before and after its approval for presbyopia. Following its approval, there was a significant increase in online searches for Vuity, with further increases observed when it became available and during Allergan’s TV commercials. The DTCA seems to have had the greatest impact on search behavior related to Vuity, rather than Vuity approval or availability. This aligns with earlier studies regarding drug searches for benign prostatic hyperplasia and thyroid eye disease [16, 17]. It seems that individuals may be more responsive to new drug advertising than to FDA approval or drug availability. We assume that this increase in search volume reflects an increasing public interest in Vuity in the United States, given the fact that at least 114 million North American people suffer from presbyopia [1].

The most popular searches related to Vuity included Vuity itself, followed by its cost, where to acquire it, and its side effects. In general, people aim to maximize their knowledge and minimize any uncertainties before trying a new drug. Therefore, not surprisingly, people not only searched Vuity itself but also searched associated terms to understand the financial and safety aspects of Vuity. One previous report has also shown a positive correlation between online searches and online purchasing behavior [18]. Thus, although patients cannot actually buy Vuity via Google shopping, the significant correlation in searches for Vuity between Google and Google Shopping may reflect people’s interest in purchasing Vuity based on their interest in treating presbyopia obtained by online searches.

This study also revealed the side effects of most concern regarding Vuity. The GEMINI 1 and GEMINI 2 studies reported headache (14.9%), conjunctival hyperemia (5.1%), vision blur (4.5%), and eye pain (4.3%) as potential side effects related to the use of Vuity, compared to rates of 7.0%, 4.0%, 0.8%, and 1.1%, respectively, in the vehicle group [2]. Therefore, we examined the search volume of Vuity and each side effect in Google Trends. None of these side effects were searched frequently in combination with Vuity; however, the term “Vuity retinal detachment” was searched frequently, with a peak on April 10–16, 2022. The peak of the search volume for Vuity itself occurred on March 27–April 2, 2022, indicating a 2-week delay in the search volume for retinal detachment. As expected, attention from the general public, ophthalmologists, and optometrists may have been focused first on the availability of Vuity, followed by subsequent public awareness of the potential risk of retinal detachment.

Although no large prospective studies have been conducted that have proven a causal relationship between topical pilocarpine and retinal detachment, numerous case reports suggest a potential increase in the risk of retinal detachment with pilocarpine eye drops for glaucoma patients, as well as with Vuity for presbyopia [5, 7, 8, 19]. The earliest description of retinal detachment in a patient with miotic medications (topical eserine) was reported by Leber in 1877 [20]. Over 30 cases of presumed miotic-related retinal detachments were reported by early 1970s [4]. After the FDA approval of topical pilocarpine in 1974, additional cases were reported from the late 1970s to early 1980s [4, 6]. A survey in 1979 found 79% of Retina Society members considered miotic medications a probable factor for retinal detachment [5]. The strong suspicion was based on retinal detachments that developed a few hours after initiation or after an increase in dosage of the miotic medication in some patients. However, some retina specialists have denied any causative role for miotic medications [5, 21, 22]. The results from Google Trends and social media including Reddit and Facebook reflect public concern about the potential risk of retinal detachment with Vuity. These findings indicated the importance of future investigation of the causal relationship between Vuity and retinal detachment. Further prospective studies and/or post-market surveillance are expected to examine whether there is a causal relationship between Vuity and retinal detachment, and if there is, to determine who is at higher risk for retinal detachment in relation to Vuity.

The geographic variation of RSV for Vuity correlated well with searches for presbyopia, indicating that, in states where Vuity has a high number of searches, presbyopia also is highly searched during the same period. The RSV for “Vuity” was highest in Wyoming, followed by North Dakota and West Virginia. We were unable to determine the reason why searches for Vuity and presbyopia are popular in these states. The prevalence of presbyopia increases with age, but the median age was not particularly high in these three states compared to other states (All States: 38.8 years, Wyoming: 38.7 years [#27 in the United States], North Dakota: 35.4 years [#47], and West Virginia:43.0 years [#3]) [23]. The proportion of people between the ages of 40 and 54 years was also not greater in these states (Wyoming: 17.5%, North Dakota: 16.4%, and West Virginia: 19.0%) compared with the average proportion in the United States (18.7%) [24]. However, according to the 2012 Fifth Edition of Vision Problems in the United States provided by the National Eye Institute and the American Health Assistance Foundation, the prevalence of hyperopia of 3 diopters or more was higher in Wyoming (10.5%), North Dakota (11.3%), and West Virginia (11.2%) compared with the overall prevalence of 9.9% in people older than 40 years old in the United States [25]. As hyperopia increases the likelihood of developing presbyopia, this may partly explain the greater interest in Vuity in these states. However, further studies are needed to clarify the significance of the geographic variation in interest in Vuity and presbyopia.

This study had several limitations. First, Google Trends provides only a relative number, but not the absolute number, for the search volume. Google Ads Keyword Planner provides the range of the absolute number, but also does not provide the absolute number. Thus, identifying the proportion of people with presbyopia who are searching Vuity in the United States is not possible. Second, although Google is a dominant search engine that comprises over 88% of desktop searches in the United States, it does not cover all the information available with other search engines or social media. A third limitation was that we could not determine if searches for Vuity were mainly driven by consumers rather than eye care providers such as ophthalmologists and optometrists. However, given the large volume of searches of 10,000 to 100,000 per month, it is unlikely that the searches are initiated by just eye care physicians. Fourth, despite our speculation that the major increase in search volume for Vuity was influenced by its approval, availability, and DTCA, an exact causal impact of these events on search behavior was difficult to prove. In addition, we do not know whether differential exposures to DTCA caused geographic variations in search volume. Lastly, a direct causal relationship between search interest and the purchase (usage) of Vuity is not clear. Nevertheless, Google Trends remains a powerful tool for evaluating public interest in and concerns on particular topics.

## Conclusion

We used Google Trends to evaluate public interest and concerns related to Vuity for presbyopia. The internet searches for Vuity significantly increased in response to its FDA approval, availability, and DTCA with TV commercials. Specific interests, other than Vuity itself, included its cost, where it can be purchased, and its side effects. Given the particular concern about retinal detachment as a potential side effect, further investigation into the causal relationship between Vuity and retinal detachment is warranted.

## References

[1] Fricke TR, Tahhan N, Resnikoff S, Papas E, Burnett A, Ho SM, et al. Global Prevalence of Presbyopia and Vision Impairment from Uncorrected Presbyopia Systematic Review, Meta-analysis, and Modelling. Ophthalmology. 2018;125: 1492–1499. doi: 10.1016/j.ophtha.2018.04.013 29753495

[2] Waring GO, Price FW, Wirta D, McCabe C, Moshirfar M, Guo Q, et al. Safety and Efficacy of AGN-190584 in Individuals With Presbyopia. Jama Ophthalmol. 2022;140: 363–371. doi: 10.1001/jamaophthalmol.2022.0059 35238902 PMC8895317

[3] Grzybowski A, Ruamviboonsuk V. Pharmacological Treatment in Presbyopia. J Clin Medicine. 2022;11: 1385. doi: 10.3390/jcm11051385 35268476 PMC8910925

[4] Pape LG, Forbes M. Retinal Detachment and Miotic Therapy. Am J Ophthalmol. 1978;85: 558–566. doi: 10.1016/s0002-9394(14)75255-9 655234

[5] Beasley H, Fraunfelder FT. Retinal Detachments and Topical Ocular Miotics. Ophthalmology. 1979;86: 95–98. doi: 10.1016/s0161-6420(79)35529-4 530570

[6] Zimmerman TJ, Wheeler TM. Side Effects and Ways to Avoid Them. Ophthalmology. 1982;89: 76–80. doi: 10.1016/s0161-6420(82)34866-6 7070779

[7] Al-khersan H, F HW Jr, Townsend JH. Retinal Detachments Associated with Topical Pilocarpine Use for Presbyopia Pilocarpine-Associated Retinal Detachments. Am J Ophthalmol. 2022. doi: 10.1016/j.ajo.2022.05.011 35609677

[8] Eton EA, Zhao PY, Johnson MW, Rao RC, Huvard MJ. Rhegmatogenous Retinal Detachment Following Initiation of Pilocarpine Hydrochloride Ophthalmic Solution 1.25% for Treatment of Presbyopia. Retin Cases Brief Reports. 2022;Publish Ahead of Print. doi: 10.1097/ICB.0000000000001309 35963010

[9] Search Engine Market Share in United State of America. Stat Counter. URL: https://gs.statcounter.com/search-engine-market-share/all/united-states-of-america.

[10] Nuti SV, Wayda B, Ranasinghe I, Wang S, Dreyer RP, Chen SI, et al. The Use of Google Trends in Health Care Research: A Systematic Review. Plos One. 2014;9: e109583. doi: 10.1371/journal.pone.0109583 25337815 PMC4215636

[11] Ayyoubzadeh SM, Ayyoubzadeh SM, Zahedi H, Ahmadi M, Kalhori SRN. Predicting COVID-19 Incidence Through Analysis of Google Trends Data in Iran: Data Mining and Deep Learning Pilot Study. Jmir Public Heal Surveillance. 2020;6: e18828. doi: 10.2196/18828 32234709 PMC7159058

[12] Mavragani A, Gkillas K. COVID-19 predictability in the United States using Google Trends time series. Sci Rep-uk. 2020;10: 20693. doi: 10.1038/s41598-020-77275-9 33244028 PMC7692493

[13] Ginsberg J, Mohebbi MH, Patel RS, Brammer L, Smolinski MS, Brilliant L. Detecting influenza epidemics using search engine query data. Nature. 2009;457: 1012–1014. doi: 10.1038/nature07634 19020500

[14] Deiner MS, McLeod SD, Wong J, Chodosh J, Lietman TM, Porco TC. Google Searches and Detection of Conjunctivitis Epidemics Worldwide. Ophthalmology. 2019;126: 1219–1229. doi: 10.1016/j.ophtha.2019.04.008 30981915 PMC7338037

[15] Skrzypczak T, Jany A, Michałowicz J, Hossa M, Bogusławska J, Targonska M. Public Interest in Cataract Surgery: Analysis and Implications of Google Trends Data from 14 European Countries. Ophthalmic Epidemiol. 2021;29: 1–8. doi: 10.1080/09286586.2021.1904513 33789529

[16] Strawbridge JC, Meer EA, Singh P, Rootman DB. Google Searches for Thyroid Eye Disease After Regulatory Approval of Teprotumumab. Jama Ophthalmol. 2022;140: 639–642. doi: 10.1001/jamaophthalmol.2022.1000 35482336 PMC9052105

[17] Skeldon SC, Kozhimannil KB, Majumdar SR, Law MR. The Effect of Competing Direct-to-Consumer Advertising Campaigns on the Use of Drugs for Benign Prostatic Hyperplasia: Time Series Analysis. J Gen Intern Med. 2015;30: 514–520. doi: 10.1007/s11606-014-3063-y 25338730 PMC4371008

[18] Farag S, Schwanen T, Dijst M. Empirical Investigation of Online Searching and Buying and Their Relationship to Shopping Trips. Transport Res Rec. 2005;1926: 242–251. doi: 10.1177/0361198105192600128

[19] Puustjärvi T. Retinal Detachment during Glaucoma Therapy. Ophthalmologica. 1985;190: 40–44. doi: 10.1159/000309490 3969260

[20] T L. Die Krakheiten der Netzhaut and der Schnerven. Handbuch der Gesammten. Augenheilkunde Leipzig: Brektkopf & Hartel, 1877.

[21] F DB, S MH. Miotic drugs, glaucoma, and retinal detachment. Mod Probl Ophthalmol. 1975; 15: 318–22. 1160910

[22] Kraushar MF, Steinberg JA. Miotics and retinal detachment: Upgrading the community standard. Surv Ophthalmol. 1991;35: 311–316. doi: 10.1016/0039-6257(91)90053-i 2011826

[23] STATSAMERICA. URL: https://www.statsamerica.org.

[24] United States Census Bureau. URL: https://www.census.gov/.

[25] 2012 Fifth Edition of Vision Problems in the U.S. URL: http://www.visionproblemsus.org/index.html.

